# Flow Stress Prediction and Hot Deformation Mechanisms in Ti-44Al-5Nb-(Mo, V, B) Alloy

**DOI:** 10.3390/ma11102044

**Published:** 2018-10-19

**Authors:** Tianrui Li, Guohuai Liu, Mang Xu, Bingxing Wang, Tianlian Fu, Zhaodong Wang, Raja Devesh Kumar Misra

**Affiliations:** 1 State Key Laboratory of Rolling and Automation, Northeastern University, Shenyang 110819, China; ltianrui21@126.com (T.L.); xumang99@126.com (M.X.); wbxang@126.com (B.W.); futianliang@126.com (T.F.); zhdwang@mail.neu.edu.cn (Z.W.); 2 Laboratory for Excellence in Advanced Steel Research, Department of Metallurgical, Materials and Biomedical Engineering, University of Texas, El Paso, TX 79968-0521, USA; dmisra2@utep.edu.cn

**Keywords:** Titanium aluminides, flow stress, microstructure, hot deformation

## Abstract

To elucidate the hot deformation characteristics of TiAl alloys, flow stress prediction, microstructural evolution and deformation mechanisms were investigated in Ti-44Al-5Nb-1Mo-2V-0.2B alloy by isothermal compression tests. A constitutive relationship using the Arrhenius model involving strain compensation and back propagation artificial neural network (BP-ANN) model were developed. A comparison of two models suggested that the BP-ANN model had excellent capabilities and was more accurate in predicting flow stress. Based on the microstructural analysis, bending and elongation of colonies, γ and B2 grains were the main microstructural constituents at low temperature and high strain rate. Dynamic recrystallization (DRX) of γ and dynamic recovery (DRY) of β/B2 were the main deformation mechanisms. With the increase of temperature and decrease of strain rate, phase transformation played an important role. The flake-like γ precipitates in B2 grains, and a coarsening of γ lamellae via α lath dissolution during compression were observed. Additionally, the flow softening process commenced with dislocation pile-up and formation of sub-grain boundaries, followed by grain refinement, twins and nano-lamellar nucleation. Continuous DRX and phase transformation promoted the formability of Ti-44Al-5Nb-1Mo-2V-0.2B alloy.

## 1. Introduction

TiAl alloys are highly promising for high-temperature structural applications in aerospace with the potential to replace nickel-based superalloys because of their excellent properties [1,2], including being of low density, having high-temperature strength, high specific modulus and creep resistance, etc. Recently, TiAl alloys were studied from the perspective of using them for turbines of aircraft engines and gas-burning power-generation plants, which are believed to be on the verge of reaching the goal for industrial scale. Unfortunately, the widespread application of TiAl alloys is limited because of their low-temperature brittleness, poor workability and narrow processing windows [3,4,5]. It is necessary to tune the thermo-mechanical processing of TiAl alloys in order to obtain better microstructure and performance [6]. However, in traditional TiAl alloys, only isothermal conditions with high manufacturing costs could be applied to prevent failure. Therefore, TNM alloys (abbreviation for Nb and Mo containing TiAl alloys) have been developed, which possess better hot workability and are suitable for conventional thermo-mechanical processing. 

TNM alloys are known to be novel multiple phase β-solidifying γ-TiAl alloys, which undergo a complicated transformation pathway during solidification because of high Nb and Mo addition [6]. Nb and Mo elements are β phase stabilizers, which significantly influence the solidification path. Thus, L→L + β→β transformation occurs that increases the amount of bcc β phase at elevated temperature and the disordered β phase, which is expected to improve the hot deformability of TNM alloys, with ordered B2 structure (B2 phase) at room temperature. The β/B2 phase affects the microstructural evolution and deformation behavior, which also depends on optimizing the deformation temperature, strain rate, and strain–stress conditions [7,8]. Additionally, the complex transformation during hot deformation of TNM alloys simultaneously affects the microstructural evolution and softening behavior. 

Further, studies of flow stress and hot deformation behavior are meaningful for TiAl alloys to determine the optimized deformation parameters, including deformation force, strain, strain rates and deformation temperatures. It may be noted that the complex non-linear relation among deformation parameters are usually described by an Arrhenius-type constitutive equation (ACE) based on dynamic material model (DMM) for TiAl alloys in many studies [9,10,11,12,13]. However, only a few studies focused on the study of flow stress of TNM alloys during hot deformation [14,15,16]. Moreover, artificial neural network (ANN) is generally considered to be an effective information treatment system which possesses self-learning ability and is more accurate and rapid than traditional constitutive equation models [17,18]. But there are almost no reports on the application of ANN model in TNM alloys. 

In this study, an Arrhenius-type constitutive model and a back propagation artificial neural network (BP-ANN) were constructed to predict the flow stress of Ti-44Al-5Nb-1Mo-2V-0.2B (at. %) alloys under hot deformation. Material constants considering the effect of strain were calculated and a comparison of models was carried out. Attention was given to microstructural evolution at different deformation conditions and operating deformation mechanisms in the studied alloy. 

## 2. Materials and Methods

The nominal composition of the TNM alloy was Ti-44Al-5Nb-1Mo-2V-0.2B (at. %). An ingot was processed by induction skull melting (ISM) under argon atmosphere of dimensions Φ110 mm × 400 mm. Subsequently, hot isostatic pressing (HIPed) was carried out for 4 h at 1260 °C and at a pressure of 175 MPa in Ar (purity: 99.999%) atmosphere, followed by furnace cooling to promote microstructural uniformity and eliminate casting defects. 

Simulated isothermal forging tests were conducted using Gleeble 3500 (DSI, St. Paul, MN, USA) at temperatures of 1050, 1100, 1150, 1200 and 1250 °C and strain rates of 1, 0.1, 0.01 and 0.001 s^−1^ in argon atmosphere. Cylindrical compressive samples of diameter 8 mm and height 12 mm were cut by electric-discharge machine (Struers, Ballerup, Denmark) perpendicular to the radial direction of the ingot in the center zone. All the specimens were heated at a rate of 10 °C/s, homogenized for 5 min at the target temperature and deformed by ~50% engineering strain. Next, they were water quenched to room temperature after completing hot compression. Between the specimen and the indenters, molybdenum disulfide and molybdenum foils were used to minimize friction during the tests. 

The deformed specimens were sectioned parallel to the compression axis for microstructural analysis. The microstructure was characterized by scanning electron microscopy (SEM, Carl Zeiss AG, Oberkochen, Germany) in back-scattered electron (BSE) mode, electron backscattered diffraction (EBSD, Carl Zeiss AG, Oberkochen, Germany) and transmission electron microscopy (TEM, FEI, Hillsboro, OR, USA) techniques. The specimens for SEM observations were mechanically polished using standard metallographic procedure, while specimens for EBSD and TEM were prepared by electron polishing and twin-jet electron polishing respectively carried out in a solution consisting of 5% perchloric acid, 35% butanol and 60% methanol (in volume fraction) at −25 °C at a voltage of 25~30 V.

## 3. Results and Discussion

### 3.1. Arrhenius Constitutive Model 

The Arrhenius constitutive equation (ACE) model is widely considered to develop a relationship between flow stress, strain rate and deformation temperature, at a certain strain during hot deformation. The equations are as follows [13,19,20]:

Z=ε˙exp[Q/(RT)]={(1)A′σn′   ασ<0.8 (for low stress level) (2)A″exp(βσ)   ασ>1.2 (for high stress level) (3)A[sinh(ασ)]n   for all σ 

where *A*, *A’*, *A’*’, *β* and *α* = *β*/*n’* are material constants, R (8.314 J mol^−1^ K^−1^) is the gas constant, *n* and *n’* are stress exponents, $\dot{\varepsilon }$ (s^−1^) is the strain rate, *Q* (kJ mol^−1^) is the effective hot deformation activation energy, *T* (K) is the absolute deformation temperature and *σ* (MPa) is the flow stress. *Z* is Zener–Holloman parameter which represents the effect of strain rate and temperature on hot deformation behavior. Equations (1)–(3) are power law function, exponential law and hyperbolic sine law equation, respectively, which are used for different stress levels. 

**Figure 1:**
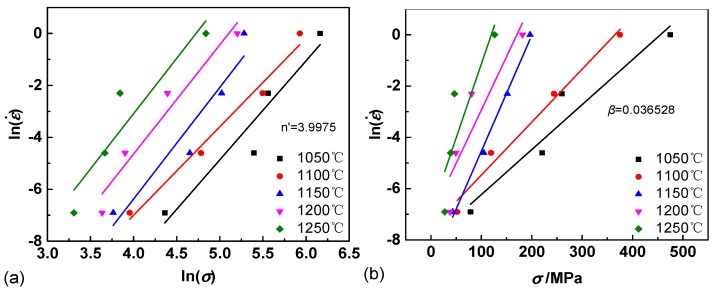

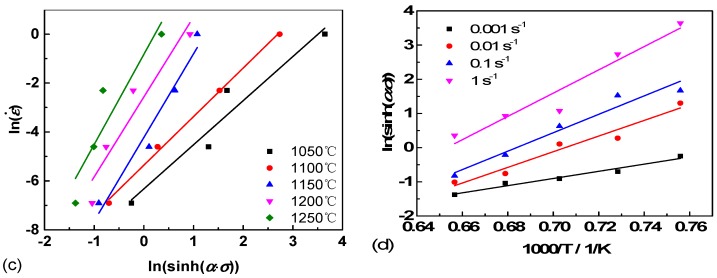
The linear fit results of (**a**) ln$\dot{\varepsilon }$-ln*σ*; (**b**) ln$\dot{\varepsilon }$ -*σ*; (**c**) ln$\dot{\varepsilon }$ -ln[sinh(*ασ*)] and (**d**) ln[sinh(*ασ*)]-1000/*T*.

It is known that strain affects the material constant. In this study, the data at true strain of 0.1 in Figure 1 was applied to calculate the material constants of the above constitutive equations. The strain effect on the material constants is presented later in the study.

Taking the natural logarithm of Equations (1)–(3), they can be expressed by:

lnZ=lnε˙+Q/RT={(4)lnA′+n′lnσ(5)lnA″+βσ(6)lnA+nln[sinh(ασ)] 

The values of *n’* (Figure 1a), *β* (Figure 1b), *n* (Figure 1c) and *Q* (Figure 1d) are calculated by the mean slope values of Figure 1 according to Equations (4)–(6) as follows:

(7) $$n^{\prime }={\left[\frac{\partial \ln\dot{\varepsilon }}{\partial \ln\sigma }\right]}_{T}\;»\;\bar{n\prime }\;=\;3.997$$

(8) $$\beta ={\left[\frac{\partial ln\dot{\varepsilon }}{\partial \sigma }\right]}_{T}\;»\;\bar{\beta }\;=\;0.037$$

(9) $$n\;=\;{\left[\frac{\partial ln\dot{\varepsilon }}{\partial \ln\left[\sin h\left(\alpha \sigma \right)\right]}\right]}_{T}\;»\;\bar{n}\;=\;2.83$$

(10) $$Q/nR={\left[\frac{\partial \ln\left[\sin h\left(\alpha \sigma \right)\right]}{\partial \left(1/T\right)}\right]}_{\dot{\varepsilon }}\;»\;\bar{Q}\;=\;555.27\mathrm{KJ}\;{\mathrm{mol}}^{-1}$$

Then, *α* = *β*/*n’* = 0.0093. Thus, the range of ασ at true of 0.1 is 0.279 to 4.185, which fits the constitutive relationship of Equation (3) and suggests that only hyperbolic sine relationship is suitable for the studied alloy. The intercept of Figure 1d, which corresponds to ln*A*, the average value of ln*A* was determined to be 42.582.

Equation (3) also can be presented as:

(11) $$\sigma \;=\;\frac{1}{\alpha }\ln\left\{{\left(\frac{Z}{A}\right)}^{\frac{1}{n}}+{\left[{\left(\frac{Z}{A}\right)}^{\frac{2}{n}}+1\right]}^{\frac{1}{2}}\right\}\;,$$

in which *Z* parameter is used to describe the flow stress. Finally, the flow stress can be described by:

(12) $$\;\sigma \;=\;\frac{1}{0.0093}\ln\left\{{\left(\frac{\dot{\varepsilon }\exp\left(555.27/RT\right)}{3.11\times 10^{18}}\right)}^{\frac{1}{2.83}}+{\left[{\left(\frac{\dot{\varepsilon }\exp\left(555.27/RT\right)}{3.11\times 10^{18}}\right)}^{\frac{2}{2.83}}+1\right]}^{\frac{1}{2}}\right\}\;$$

**Figure 2:**
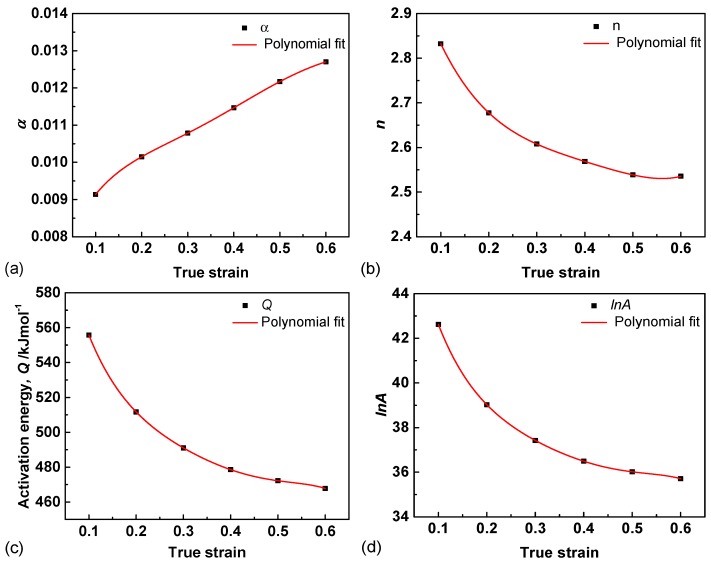
Polynomial relations of (**a**) *α*; (**b**) *n*; (**c**) *Q* and (**d**) ln*A* to true strains.

Using the same solution, the calculation results of *α*, *n*, *Q* and ln*A* values under different strains ranging from 0.1 to 0.6 with an interval of 0.1 are shown in Figure 2. 

Based on the stress exponent (*n*) values that vary from 2.83 to 2.54, it can be deduced that grain boundary and dislocation sliding may jointly dominate the controlled mechanism during hot deformation [5,21]. As the deformation proceeds, DRX occurs primarily by means of dislocation slip and substructure. With strain to 0.6, more dynamic recrystallized grains nucleate and grain boundary sliding may play a more important role in further deformation, leading to a decrease of *n*. 

Moreover, the activation energy (*Q*) can also influence the deformation mechanism. The calculated *Q* values decreased from 555.74 to 467.81 kJ mol^−1^, as shown in Figure 2c. The lower *Q* suggests that the alloy can deform more easily, because it needs lower external energy to activate dislocations and form substructures. Thus, it may be easier for deformation and flow softening if the alloys are deformed at a higher true strain. 

Based on the square of correlation coefficient (R^2^ = 1) values, the fifth order polynomial may precisely represent the material constants. The polynomial coefficient can be obtained and the functions for Figure 2 are presented as follows:

(13) $$\;\alpha \;=\;0.0066+0.039\varepsilon -0.18\varepsilon^{2}+0.45\varepsilon^{3}-0.53\varepsilon^{4}+0.23\varepsilon^{5}\;$$

(14) $$\;n\;=\;3.15-4.26\varepsilon +12.59\varepsilon^{2}-16.05\varepsilon^{3}+2.50\varepsilon^{4}+7.09\varepsilon^{5}\;$$

(15) $$\;\ln A\;=\;51.88-141.32\varepsilon +623.29\varepsilon^{2}-1545.54\varepsilon^{3}+1978.35\varepsilon^{4}-1007.19\varepsilon^{5}\;$$

(16) $$\;Q\;=\;667.01-1696.32\varepsilon +7491.36\varepsilon^{2}-18879.10\varepsilon^{3}+24619.21\varepsilon^{4}-12745.14\varepsilon^{5}\;$$

**Figure 3:**
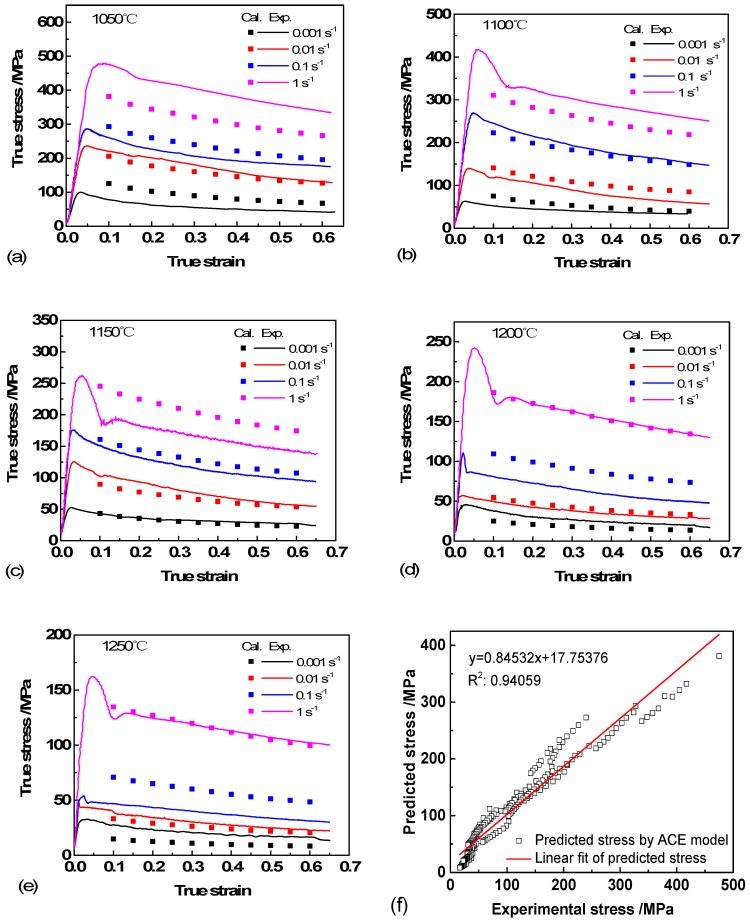
Comparison of stress–strain curves between tests and Arrhenius constitutive model at different temperatures and strain rates: (**a**) 1050 °C; (**b**) 1100 °C; (**c**) 1150 °C; (**d**) 1200 °C; (**e**) 1250 °C; (**f**) correlation analysis.

Substituting Equations (13)–(16) into Equation (11), the plot of flow stress at a certain temperature and strain rate is shown in Figure 3. The square of correlation coefficient (R2) value of the ACE was 0.94059. Clearly, the flow behavior of Ti-44Al-5Nb-1Mo-2V-0.2B alloy can be predicted by this model and has a good agreement with the experimental data at certain deformation conditions. However, it can be observed that the model has an obvious error at high strain rates, especially when the temperatures are low, and this is consistent with the previous studies [22,23]. 

### 3.2. Artificial Neural Network Model

Artificial neural network (ANN) is a structure mimicking biological neural connection which could be applied to designing pattern analysis, signal processing etc. [15,16]. The model utilizes knowledge from outside, for instance, sample data. With its analytical and learning capability, the variation in flow stress, microstructure and mechanical properties can be studied. 

**Figure 4:**
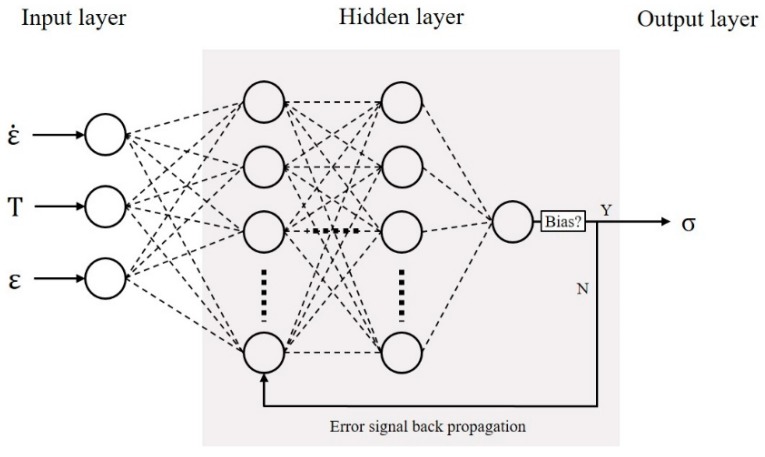
The schematic of ANN model.

The ANN model is usually divided into three parts to predict flow stress (σ) during hot deformation, as shown in Figure 4, including the input layer, hidden layer and output layer. The circle in the schematic represents the different basis unit: neuron. All the neurons are independent of each other within a layer, while each layer is complicatedly connected. 

**Figure 5:**
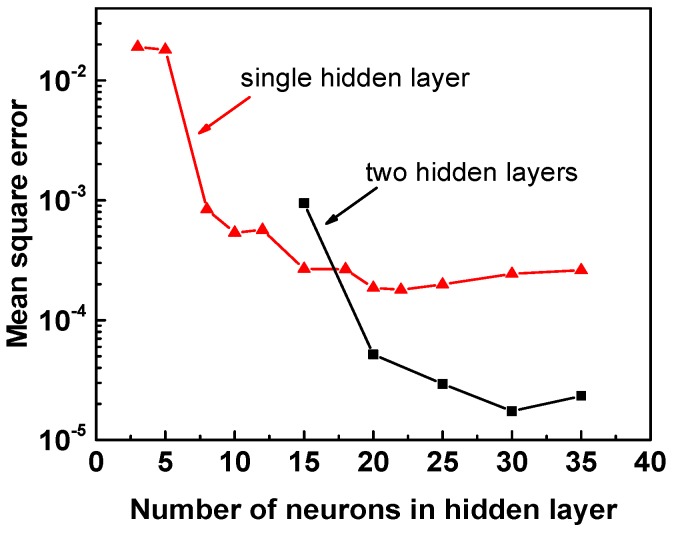
Dependence of mean square error and number of layers and neurons in hidden layer.

For better accuracy of the ANN model, it is necessary to analyze the dependence of mean square error (MSE) versus neuron numbers in the hidden layer, as shown in Figure 5. The red line represents the MSE for a single hidden layer, which is almost constant when neurons are more than 15. The black line represents the MSE for two hidden layers, in which there are 10 neurons in the second layer. It may be noted that the two hidden layers can lower the MSE by more than one order of magnitude and that MSE would reach the minimum at 30 neurons in the first layer. Thus, a 3-(30 × 10)-1 mode network was selected, which means there are two hidden layers belonging to 30 neurons and 10 neurons, respectively. 

Selecting 80% data from all the information data to train the network, and the remaining 20% are test data for validating whether the ANN could deal with complicated and highly non-linear relationship between variables.

Before adopting the network, all data should be normalized to [−1, 1] avoiding systematic error accumulation by employing the method as:

(17) $$\;\hat{x}\;=\;2\frac{x-x_{\min}}{x_{\max}-x_{\min}}-1\;$$

where *x* is the experimental value, $\hat{x}$ is the normalized value of *x*, $x_{\max}$ and $x_{\min}$ are the maximum and minimum values of *x*, respectively. 

In the back-propagation (BP) network, three input hot processing parameters: strain rate ($\dot{\varepsilon }$), deformation temperature (*T*) and true strain (*ɛ*), are sent to the input layer. The BFGS (Broyden Fletcher Goldfarb Shanno)–Quasi Newton training (Trainbfg) algorithm, which is widely regarded to be suitable for small-size networks, was used during the data training to modify the weight and bias until they fit the requirement. Otherwise, learning efficiency is also an important factor for learning process. In general, a smaller learning efficiency could further enhance the ANN network stability. In this work, it is selected as 0.05 considering the computational efficiency. 

**Figure 6:**
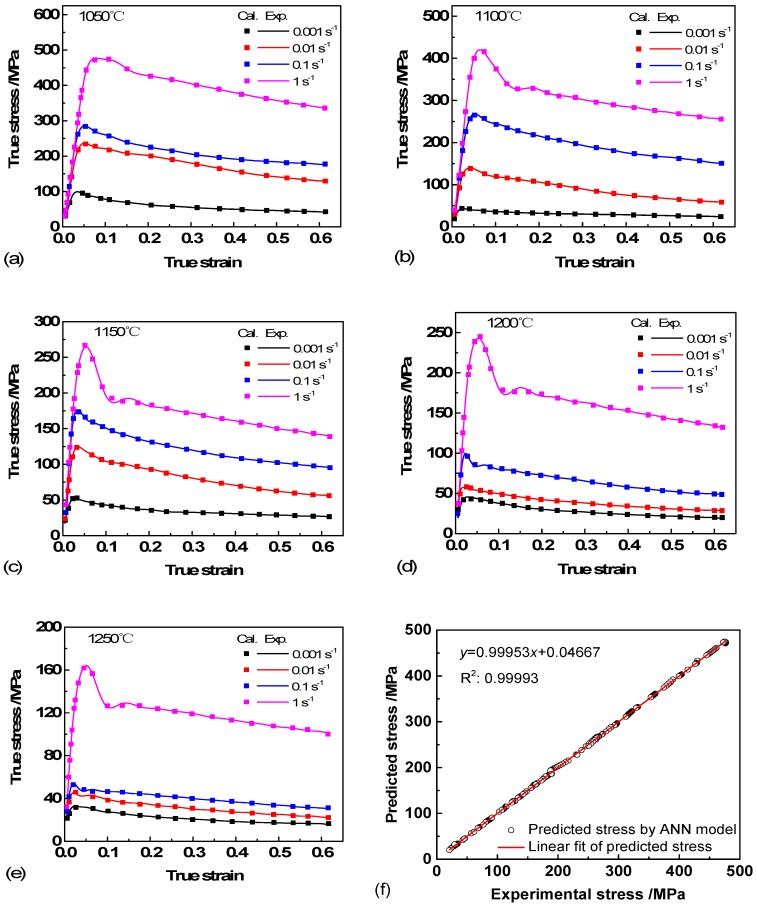
Comparison of the experimental and predicted data of Ti-44Al-5Nb-1Mo-2V-0.2B alloy: (**a**) 1050 °C; (**b**) 1100 °C; (**c**) 1150 °C; (**d**) 1200 °C; (**e**) 1250 °C; (**f**) correlation analysis.

The un-normalized output variable flow stress (*σ*) is carried out after the neural network is trained, tested and simulated. Direct comparison of calculated (Cal.) and experimental (Exp.) data are shown in Figure 6a–e. It can be seen that good performance of network is achieved. 

Figure 6f is the linear regression between ANN predicted stress and 20% test experimental data. The R^2^ value is 0.99993, which indicates that the proposed ANN model can provide more accurate evaluation of flow stress during hot deformation than Arrhenius constitutive model (Figure 3) [24]. 

### 3.3. Microstructural Evolution and Deformation Mechanisms

As shown in Figure 6, it can be seen that the peak stress was up to 480 MPa when the temperature was 1050 °C and strain rate of 1 s^−1^. Whereas, the peak stresses were only 162 MPa and 99 MPa when the specimens were deformed at 1250 °C/1 s^−1^ and 1050 °C/0.001 s^−1^. The former is ≈3 times and 5 times of the latter, which shows that the flow stresses decrease significantly with increased deformation temperature and decreased strain rate. All the flow stress curves are characteristic of TiAl alloys that increase sharply to a peak stress at the beginning and then decrease gradually to a steady state with increasing strain. 

Under certain deformation conditions, Ti-44Al-5Nb-1Mo-2V-0.2B alloy shows discontinuous yielding phenomenon (DYP) [25,26]. The flow stress increases rapidly to the upper yield stress (the first peak stress) point, and then drops sharply to the lower yield stress point followed by a second peak stress with increase of strain. The fluctuations are more prominent at higher temperature and strain rate. The occurrence of DYP is closely related to the hot deformation softening mechanism of our TiAl alloy. The complicated interactions of work hardening, DRY and DRX under different deformation conditions led the flow stress to fluctuate to different degrees. The behavior is attributed to the interaction of dislocation slip and twinning. Such behavior also suggests the occurrence of elongation of γ, B2 and lamellar colonies, the formation of subgrains and the decomposition of lamellae during the initial period of deformation. 

**Figure 7:**
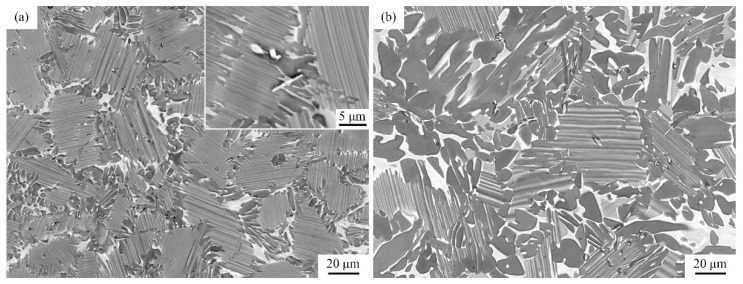
Microstructure of cast Ti-44Al-5Nb-1Mo-2V-0.2B alloy: (**a**) as-cast and (**b**) as-HIPed.

Figure 7 shows the microstructure of Ti-44Al-5Nb-1Mo-2V-0.2B alloy before hot compression. The as-cast microstructure mainly consisted of α2/γ lamellar colonies, which are surrounded by equiaxed γ (grey) and irregular B2 grains (bright), as shown in Figure 7a. A few Ti(B, V) granular and needlelike inclusions are distributed at colony boundaries, as shown in the inset. 

The microstructure of fine α2/γ lamellar colonies (with grain size of 30–40 μm) and mixture of γ and B2 at boundaries are because of the solidification path and refinement effect of Ti(B, V). During solidification, the liquid phase transforms into β phase, which is ascribed to high content of β stabilizers (Nb, Mo), namely complete β phase solidification, accompanied by the precipitation of Ti(B, V). First, β phase may be refined by Ti(B, V). Next, α phase forms from primary β grains by ${\left\{0001\right\}}_{\alpha }∥{\left\{1\bar{1}0\right\}}_{\beta }$ and $11\bar{2}0_{\alpha }∥{\langle 111\rangle }_{\beta }$ orientations and partitions β grains into different variants, i.e., primary β grain divides into different α grains with no more than 12 orientations [1]. At the same time, Ti, Nb and Mo elements diffuse to α (Al-rich) grain boundaries and promote β/B2 phase formation during subsequent solidification process. On further cooling, γ phase finally precipitates from α/α2 and β/B2 grains forming lamellar colonies and grain boundary γ and B2 phase. 

Figure 7b shows the microstructure after HIPed treatment at 1260 °C. It can be seen that α2/γ lamellar colonies are coarsened and partly decomposed, along with the coarsening of lamellar spacing and grain boundary γ and B2 phase. This microstructural morphology arises because of holding above the eutectoid transformation temperature for about 4 h and is generally supposed to be more easily deformed and softened during subsequent deformation. 

**Figure 8:**
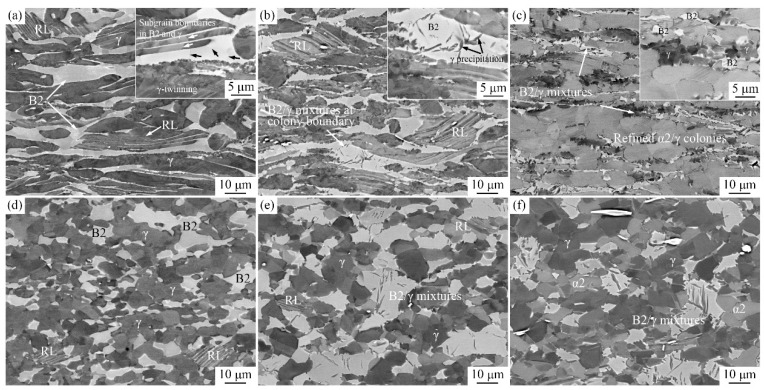
Microstructure of Ti-44Al-5Nb-1Mo-2V-0.2B alloy deformed at (**a**) 1050 °C/0.1 s^−1^; (**b**) 1150 °C/0.1 s^−1^; (**c**) 1200 °C/0.1 s^−1^; (**d**) 1050 °C/0.001 s^−1^; (**e**) 1150 °C/0.001 s^−1^; (**f**) 1200 °C/0.001 s^−1^.

The typical microstructure after hot deformation at different temperatures and strain rates for 50% engineering strain of the alloy are shown in Figure 8. Figure 8a shows the microstructure deformed at 1050 °C/0.1 s^−1^ in (α2 + γ + B2) phase region. Lamellar colonies, γ and B2 phases are severely deformed and elongated perpendicular to the compression direction. The γ and B2 grains around elongated colonies are suggested to coordinate α2/γ lamellae deformation and ensure that the alloy does not crack. A few subgrain boundaries in both γ and B2 phases and twins in γ grains can be observed in the inset image of Figure 8a. The size of γ and B2 subgrains are ~2 μm and 5 μm, respectively. The deformation of lamellar colonies is large, which can provide sufficient driving force for DRX and phase transformation, thereby resulting in a small amount (~12.17% in volume fraction) of remnant lamellae (RL). DRX prefers to occur at colony boundaries. The γ laths in RL are coarse and α2 laths are relatively thin, because of the occurrence of α to γ + B2 transformation. 

In Figure 8b, the microstructure deformed at 1150 °C/0.1 s^−1^ continued to be elongated. The phase volume fraction of lamellar colonies, γ and B2 were 25.25%, 45.34% and 29.41%, respectively. The content of α2/γ colonies was increased and α2 laths in RL were coarsened, when comparing with Figure 8a, whereas γ lamellae become narrow. It is considered that with the increase of deformation temperature, γ to α transformation is activated and the phase contents are thus changed. Moreover, γ + B2→γ + β transition also occurred. This means that γ phase transformed into β phase, which led to decreased content of γ phase at grain boundaries. Because of the fast cooling rate (water quenched), the supersaturated Al in β phase may precipitate as flake-like γ grains, as shown in the inset of Figure 8b. The content of lamellar colonies was further increased to 76.31% when the temperature was 1200 °C. Many equiaxed lamellar colonies, with an average size of 7.2 μm were generated (Figure 8c). The thickness of the laths, which is nano scale, is so thin that it was hardly observed by SEM. The content of γ and B2 are apparently decreased to 12.80% and 10.89%. According to the observations, 1200 °C may be close to the single α phase region. However, because of the stabilization effect of Nb and Mo, B2/γ mixture at the colony boundaries continued to exist. The elimination of the B2/γ mixture by heat treatment is relatively difficult and complex, as reported previously [27,28]. 

As the strain rate decreases to 0.001 s^−1^, completely DRXed grains were observed in the deformed specimens. The content of α2/γ lamellar colonies was ~5.16% and 2.43% at 1050 °C and 1150 °C, respectively. A few RL could be observed, as shown in Figure 8d,e, because of the complete breakdown of lamellae after holding for a long time at the deformation temperature. No RL was observed in Figure 8f, while the content of α2 phase was 21.48%. The microstructure deformed at 0.001 s^−1^ finally transformed into a mixture of equiaxed γ, α2 and B2 grains. The mean size of γ grains and the amount of γ precipitation from B2 grains increased significantly with increased temperature, whereas B2 grains showed a decreasing trend. These results indicate that during the deformation, the low strain rate facilitates DRX and growth of γ, α2 and B2 grains as well as γ to β transition at higher temperatures. 

Based on the above analysis of typical deformation microstructure, it can be concluded that when the temperature is below 1150 °C and the strain rate is high, the bending and elongation of colonies, γ and B2 grains are the main microstructural constituents. DRX of γ and DRY of β/B2 occurs simultaneously. When the temperature is greater than 1150 °C, phase transformation plays a more important role, namely with a high temperature and low strain rate, which needs more incubation time and driving force. 

**Figure 9:**
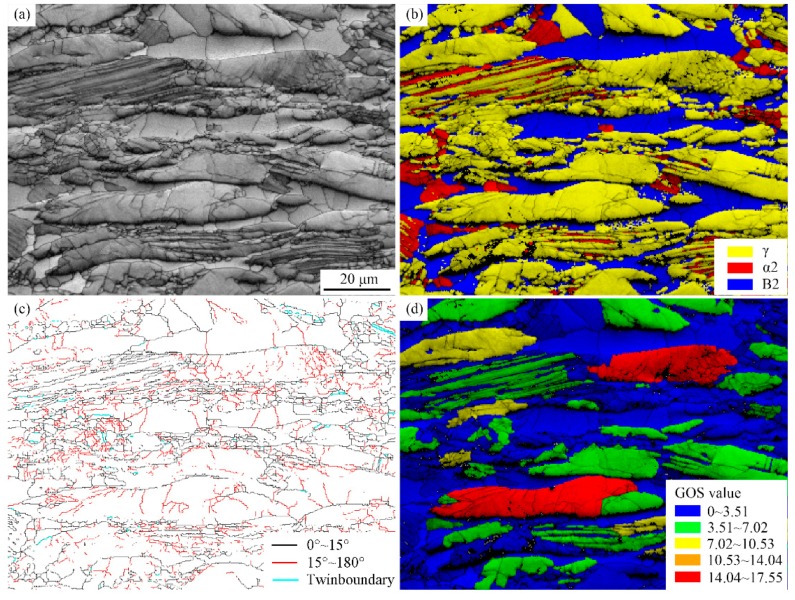
The EBSD analysis of Ti-44Al-5Nb-1Mo-2V-0.2B alloy deformed at 1050 °C/0.1 s^−1^ at strain of 50%: (**a**) Image quality (IQ) map; (**b**) phase distribution map; (**c**) grain boundary (GB) map; (**d**) grain orientation spread (GOS) map.

The microstructure deformed at 1050 °C/0.1 s^−1^ at a strain of 50% was further illustrated by EBSD analysis using a 0.2 μm beam step, as shown in Figure 9. Multiple morphologies were observed in Figure 9a, including elongated α2/γ lamellae, γ and B2 grains, formation of subgrain boundaries and the breakdown of lamellae. The deformed microstructure mainly consisted of γ grains (62.2%) and some B2 phases (22.9%), plus a few α2 (9.2%) phases, as shown in Figure 9b. During the deformation of TiAl alloys [29], DRX of γ phase tends to occur because of its low stacking fault energy, while recovery is the main mechanism for the B2 phase, with relatively high stacking fault energy. The α2/γ lamellar colonies were slightly decomposed and thin α2 laths were observed, indicating the occurrence of DRX for γ laths and α to γ transition [30]. In Figure 9c, the volume fraction of high angle grain boundaries (HAGBs, 15°~180° in black lines) and low angle grain boundaries (LAGBs, 0°~15° in red lines) were 33.4% and 66.6%, respectively. In this study, high content of LAGBs indicated that DRY occurred both in γ and B2 grains when the alloy was deformed at 1050 °C. LAGBs generally suggest that substructure formation occurs during deformation, which is obtained by the pile-up and rearrangement of dislocations. The volume fractions of LAGBs generally increase with the increase of distortion energy [31]. Moreover, some mechanical twin boundaries (2.4%) were observed in deformed γ grains (60° in blue lines). The GOS map in Figure 9d indicates that the bending lamellar and some deformed γ grains had high local strain with high dislocation density and distortion energy (red and green color region), while the equiaxed grains region had low distortion because of stress release through phase transition and DRX. Furthermore, the severe deformation resulted in different degrees of deformation of lamellar colonies, which are visible at the bottom of Figure 9d. 

**Figure 10:**
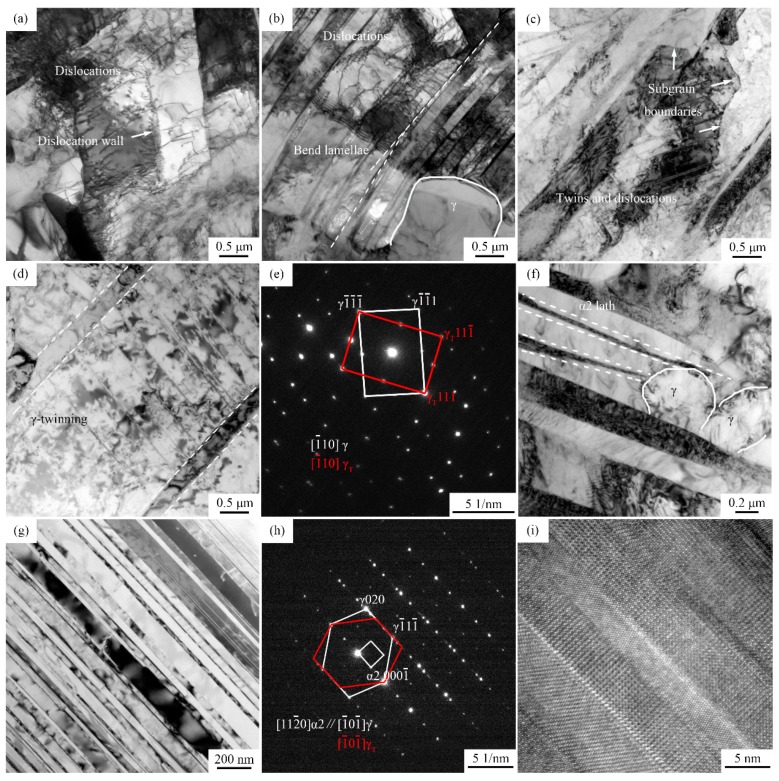
TEM analysis of Ti-44Al-5Nb-1Mo-2V-0.2B alloy deformed at 1200 °C/0.1 s^−1^ at strain of 50%: (**a**) dislocations in γ grains; (**b**) fragmented lamellae; (**c**–**e**) mechanical twins in γ laths and the selected area electron diffraction (SAED) pattern; (**f**) decomposition of α2 laths; (**g**,**h**) the bright-field image of nano α2/γ lamellae and its SAED pattern; (**i**) the high-resolution transmission electron microscopy (HRTEM) image of nano α2/γ lamellae.

The TEM images in Figure 10 reveal the deformed microstructure of Ti-44Al-5Nb-1Mo-2V-0.2B alloy compressed at 1200 °C, 0.1 s^−1^ to 50% strain. The typical recrystallization microstructure and phase transformation process can be briefly described. 

High density of dislocations in γ grains and irregular lamellae is a main feature of the alloy, which can accelerate the formation of dislocation walls, fragmented lamellar colonies and sub-grain boundaries [32], as shown in Figure 10a–c. Some fine dislocation-free DRX γ grains of diameter ~1 μm were observed (Figure 10b). Furthermore, decomposition of thin α2 laths, coarsening of γ lamellae and formation of fine γ grains in broken lamellae are shown in Figure 10d,f. As shown above, the decomposition of α2/γ lamellar colonies is induced by the combined effect of dislocation pile-up and α2 to γ transformation, which is generally determined by the intrinsic nature of the alloy [33,34]. 

As we know, γ-TiAl is a tetragonal L10 ordered structure with *c*/*a* = 1.02, in which Ti and Al atoms occupy (002) planes alternately [35]. However, the difference between *c* and *a* axis is so small that TiAl can be approximately regarded as a face centered cubic (fcc) structure. During plastic deformation of γ-TiAl (elongation and bending of γ grains and α2/γ lamellar colonies), the slip occurs mainly by 1/2〈110〉 ordinary dislocations, 〈101〉 and 1/2〈112〉 super dislocations on {111} plane at high temperatures [36]. High density of dislocations leads to high stored energy in γ grains and α2/γ lamellar interfaces, which annihilate and rearrange, leading to the formation of dislocation walls and sub-boundaries. However, Nb and Mo are difficult to diffuse, such that the movement of dislocations is slow and the effect of the DRY process is weak [37]. Three Shockley partial dislocations $1/6\langle 11\bar{2}\rangle$ can be dissociated from b = 1/2〈110〉 ordinary dislocations, which promote the shear of TiAl matrix to twinning orientation, resulting in γ stacking faults (SF) and mechanical twins, as shown in Figure 10c,d. Figure 10e shows the selected area electron diffraction (SAED) pattern. It seems that the majority of the twins originate from lamellar interface in γ laths, which would segment the lamellae [38]. Besides, nano-lamellae as well as twins with twin boundaries parallel to the lamellar interface are also observed in Figure 10g. The orientation relationships of γ twins and nano α2/γ lamellae were confirmed, as illustrated by the SAED technique in Figure 10h. Figure 10i shows the high-resolution transmission electron microscopy (HRTEM) image of α2/γ laths with width of 5~10 nm (the incident beam parallel to the axis $\left[\bar{1}0\bar{1}\right]$γ//$\left[11\bar{2}0\right]$α2).

Higher additions of Nb and Mo alloying elements significantly reduce the stacking fault energy of γ-TiAl. The reaction of Shockley dislocation formation is more common, for instance, $1/2\left[\bar{1}10\right]\to 1/6\left[\bar{1}2\bar{1}\right]+1/6\left[\bar{2}11\right]+\mathrm{SF}$. Planar defects and mechanical twinning are consequently activated [39]. 

Microstructural transformations are generally thermally activated processes and are complicated for Ti-44Al-5Nb-1Mo-2V-0.2B alloy. According to the phase diagram of TNM alloys [40], 1200 °C is in the α + γ + β/B2 phase region and slightly higher than the eutectoid transformation (α→β/B2 + γ) temperature, neglecting the α→α2 and B2→β transition. During deformation, α→β/B2 + γ or α→γ proceeds. This means that α phase would decompose to β/B2 and γ phase, as expressed in Figure 10b, 10d and 10f, γ laths coarsen or γ and β/B2 grains grow where the continuities of lamellae are interrupted and stress relaxations take place. Moreover, the transition can be a reverse process that DRXed β/B2 and γ phase may also transform into α phase, caused by the low strain rate (sufficient transition time) and localized stress concentration. Then, the multiple-phase structure (retained α2/γ lamellae, recrystallized α, γ and B2 grains) would be obtained. 

**Figure 11:**
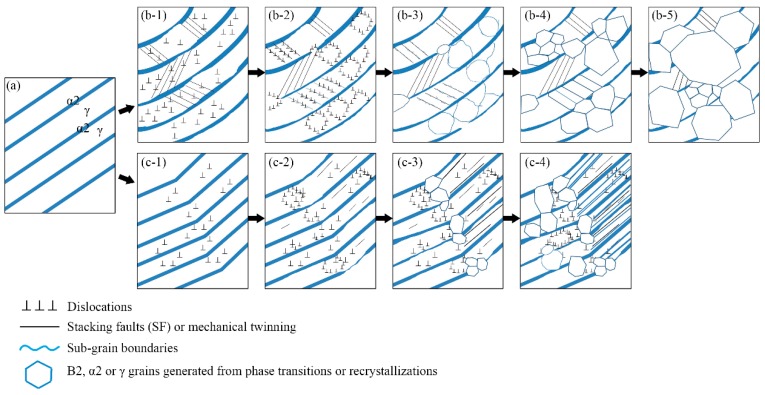
Schematic illustration showing the deformation processes of α2/γ lamellar colonies in Ti-44Al-5Nb-1Mo-2V-0.2B alloy.

Based on the above observation, the schematic illustration of the decomposition processes of α2/γ lamellae can be deduced, as shown in Figure 11. Figure 11a illustrates the morphology of α2/γ lamellae. During the initial deformation period, the lamellae are bent (Figure 11(b-1)) or kinked (Figure 11(c-1)) accompanied by abundant dislocations and α2 lath dissolution. Mechanical twins generate from α2/γ interface with a certain angle and end at another adjacent lamellar interface [41]. As the strain is continuously increased, large amounts of dislocations pile up and tangle at twin boundaries. Dislocations then rearrange and annihilate, leading to the formation of dislocation walls and sub-grain boundaries, as shown in Figure 11(b-2,b-3). With the progress in deformation, sub-grain boundaries as well as twin boundaries may transform into HAGBs (Figure 11(b-4)). The laths are divided into refined grains that grow and finally break the lamellar structure (Figure 11(b-5)). The other case is that SFs and mechanical twins may originate parallel with the α2/γ interface [42], as is clearly shown by lines in Figure 11(c-2). B2, α2 and γ grains generated from phase transformation and DRX preferentially occur at kinked positions (Figure 11(c-3)). Meanwhile, a number of SFs and twins slip and extend inducing the precipitation of α2 or γ plates. Finally, refined lamellae or nano α2/γ lamellar configuration can be formed, as is illustrated in Figure 11(c-4). 

DRY, continuous DRX and phase transition are flow softening mechanisms in Ti-44Al-5Nb-1Mo-2V-0.2B alloy. The flow softening process begins with dislocation pile-up and formation of sub-boundaries, followed by sub-boundaries and twin-induced DRX process as well as repeated phase transformation. 

## 4. Conclusions

In this study, the Arrhenius constitutive model (ACE) and Artificial neural network model (ANN) were adopted to predict the flow behavior. Microstructural characteristics at 1050–1250 °C and 1–10^−3^ s^−1^ were studied to demonstrate the deformation mechanisms in Ti-44Al-5Nb-1Mo-2V-0.2B alloy. The following are the conclusions: 

(1) The true-stress–true-strain curves of Ti-44Al-5Nb-1Mo-2V-0.2B alloy deformed in the temperature range of 1050–1250 °C and in the strain rate range of 1–10^−3^ s^−1^ showed characteristics of work hardening, discontinuous yielding phenomenon and flow softening. Interactions between work hardening, DRY and DRX under different deformation conditions led to fluctuations in flow stress to different degrees. 

(2) The ACE, considering the effect of strain and ANN, including two hidden layers with 30 neurons and 10 neurons, was developed to predict flow stress of Ti-44Al-5Nb-1Mo-2V-0.2B alloy. Comparing the two models, the prediction of the ANN model was superior to the ACE model and the square of correlation coefficient (R^2^) of ANN model was 0.99993. 

(3) When the compression temperatures were less than 1100 °C and strain rates were high, the elongation of lamellar colonies, γ and B2 grains were the primary deformed microstructure. With an increase of temperature and decreasing strain rates, especially when the temperature was greater than 1200 °C, phase transformation played an important role. 

(4) DRY, continuous DRX and phase transition are flow softening mechanisms in Ti-44Al-5Nb-1Mo-2V-0.2B alloy. Dislocation pile-up and rearrangement as well as the formation of mechanical twins promoted the DRX process. Nano-lamellae with lath width of 5~10 nm were also observed. Phase transformation of α to (β/B2 + γ) phase and DRX of γ phase led to the decomposition of lamellar colonies.
